# Effects of transthoracic echocardiography on the prognosis of patients with acute respiratory distress syndrome: a propensity score matched analysis of the MIMIC-III database

**DOI:** 10.1186/s12890-022-02028-5

**Published:** 2022-06-25

**Authors:** Daoran Dong, Yan Wang, Chan Wang, Yuan Zong

**Affiliations:** Department of ICU, Shaanxi Provincial People’s Hospital, No. 256, Youyi West Road, Beilin District, Xi’an, Shaanxi China

**Keywords:** Echocardiography, Acute respiratory distress syndrome, Critical care, Prognosis

## Abstract

**Background:**

Acute respiratory distress syndrome (ARDS) has high mortality and is mainly related to the circulatory failure.Therefore, real-time monitoring of cardiac function and structural changes has important clinical significance.Transthoracic echocardiography (TTE) is a simple and noninvasive real-time cardiac examination which is widely used in intensive care unit (ICU) patients.The purpose of this study was to analyze the effect of TTE on the prognosis of ICU patients with ARDS.

**Methods:**

The data of ARDS patients were retrieved from the MIMIC-III v1.4 database and patients were divided into the TTE group and non-TTE group. The baseline data were compared between the two groups. The effect of TTE on the prognosis of ARDS patients was analyzed through multivariate logistic analysis and the propensity score (PS). The primary outcome was the 28-d mortality rate. The secondary outcomes included pulmonary artery catheter (PAC) and Pulse index continuous cardiac output (PiCCO) administration, the ventilator-free and vasopressor-free days and total intravenous infusion volume on days 1, 2 and 3 of the mechanical ventilation. To illuminate the effect of echocardiography on the outcomes of ARDS patients,a sensitivity analysis was conducted by excluding those patients receiving either PiCCO or PAC. We also performed a subgroup analysis to assess the impact of TTE timing on the prognosis of patients with ARDS.

**Results:**

A total of 1,346 ARDS patients were enrolled, including 519 (38.6%) cases in the TTE group and 827 (61.4%) cases in the non-TTE group. In the multivariate logistic regression, the 28-day mortality of patients in the TTE group was greatly improved (OR 0.71, 95%CI 0.55–0.92, *P* = 0.008). More patients in the TTE group received PAC (2% *vs.* 10%, *P* < 0.001) and the length of ICU stay in the TTE group was significantly shorter than that in the non-TTE group (17d vs.14d, *P* = 0.0001). The infusion volume in the TTE group was significantly less than that of the non-TTE group (6.2L vs.5.5L on day 1, *P* = 0.0012). Importantly, the patients in the TTE group were weaned ventilators earlier than those in the non-TTE group (ventilator-free days within 28 d: 21 d *vs*. 19.8 d, respectively, *P* = 0.071). The Kaplan–Meier survival curves showed that TTE patients had significant lower 28-day mortality than non-TTE patients (log-rank = 0.004). Subgroup analysis showed that TTE after hemodynamic disorders can not improve prognosis (OR 1.02, 95%CI 0.79–1.34, *P* = 0.844).

**Conclusion:**

TTE was associated with improved 28-day outcomes in patients with ARDS.

**Supplementary Information:**

The online version contains supplementary material available at 10.1186/s12890-022-02028-5.

## Introduction

Acute respiratory distress syndrome (ARDS) is associated with adverse clinical outcomes and has an approximate overall mortality rate of 40%, despite the most standard treatment. ARDS patients have been shown to have a good tolerance to relative hypoxemia. However, their survival rate is not necessarily increased by the improvement of oxygenation [1]. ARDS patients frequently suffer from circulatory failure, which is independently related to death [2] and often accompanied by hemodynamic instability, such that more than 60% of the patients suffer from hemodynamic disorders, among which 65% need to use catecholamine drug [3, 4]. ARDS causes shock according to the following three factors: (1) pulmonary arterial pressure (PAP) elevation caused by vasoconstriction due to microthrombosis, arterial remodeling and hypoxia, acidosis and/or inflammatory mediators; (2) the impact of mechanical ventilation on the function of the right ventricle (RV); (3) RV failure caused by abnormal tissue oxygen demand and hemodynamic disorders due to sepsis, which probably results from insufficient preload or excessive afterload. Assessing the volume status and correcting hemodynamic disorders helps to avoid the second attack based on hypoxemia and represents an important part of the ARDS treatment [5].

Hemodynamic monitoring of ARDS patients contributes to developing reasonable therapeutic regimens and improving the prognosis. There are many methods for hemodynamic monitoring and evaluation of heart function, among which pulse index continuous cardiac output (PiCCO), pulmonary artery catheter (PAC) and echocardiography are the three most commonly used methods and help to guide treatment adjustments. However, the invasive nature of monitoring and the requirements for equipment, technology, and operators severely limit the PiCCO and PAC clinical application, while there are also hidden dangers of complications such as blood flow infection, arrhythmia, pulmonary embolism, pulmonary arterioles rupture and hemorrhage, airbag rupture, catheter knot, etc. during the placement of the catheter, and are expensive. Moreover, studies have shown that PiCCO-based fluid management does not improve outcomes compared to central venous pressure (CVP)-based fluid management [6]. Likewise, a prospective cohort study have shown PAC was even associated with increased mortality and increased utilization of resources [7]. Echocardiography, a minimally invasive and repeatable hemodynamic monitoring tool, has become increasingly essential in the management of ARDS because it can not only help differentiate the causes of shock, but it can also provide real-time information of volume status and cardiac function. It is the best bedside method to repeatedly assess cardiac function, and the rapid progress in ultrasonic techniques for critical diseases has made it possible to achieve early diagnosis and prognostic evaluation of ARDS patients. Doctors can judge the disease severity and promptly adjust the treatment plan with the help of transthoracic echocardiography (TTE).

Despite these advances, the rationality of TTE for ARDS patients has not yet been assessed, and active adjustment of the treatment plan based on the results of ultrasonography is not always achieved by doctors [8]. In addition, preoperative echocardiography was found unable to reduce mortality and the length of stay for patients undergoing non-cardiac surgery [9]. Some studies also suggested that TTE cannot improve the Acute Physiology and Chronic Health Evaluation II(APACHEII) score for the risk of death in critically ill patients [10].

In this study, we perform a retrospective analysis to observe whether TTE can affect the short-term prognosis and related indexes of intensive care unit (ICU) patients with ARDS. This work provides a definite basis for the rational use of TTE in clinical practice, reducing the overuse of it and optimizing the allocation of medical resources.

## Methods

### Study population

The data in this study came from the MIMIC-III database. MIMIC-III is an open access medical database, jointly released by the Massachusetts Institute of Technology(MIT) Laboratory for Computational Physiology, Beth Israel Deaconess Medical Center, Philips Medicine and the National Institutes of Health [11]. It contains the hospitalization information of more than 50,000 patients admitted to Beth Israel Deaconess Medical Center from June 2001 to October 2012, including the vital signs, drugs, laboratory test results, clinical observation results, records made by nurses, fluid balance, imaging reports, length of stay and survival data.

All the patients in the database were screened. Inclusion criteria were as follows: (1) adults aged ≥ 18 years old, with complete medical records, including ultrasound reports during the ICU stay; (2) Length of ICU stay ≥ 72 h; (3) Meeting the Berlin criteria for ARDS [12]. ARDS is defined as follows: acute attack, oxygenation index ≤ 300 mmHg, bilateral infiltration on chest X-ray and which the lung edema pattern could not be explained by heart failure or fluid overload. We use the following coding book of the database to define ARDS population that met Berlin definition: (1) Onset of ARDS is acute—we assume this as our cohort is only recently mechanically ventilated patients (i.e. exclude trach) (2) Bilateral opacities—we parse the free-text radiology reports for mention of opacities/infiltrates (3) PEEP > 5cmH2O. To exclude cardiogenic lung edema, we first excluded patients with heart failure on admission, and then use the coding book of the database to extract the information about ARDS patients, including echocardiography, and reviewed. Based on the oxygenation index, ARDS is classified into mild, moderate and severe degrees. For patients admitted to the ICU several times, only the data related to the first ICU admission were considered.

### Data extraction

**Fig. 1:**
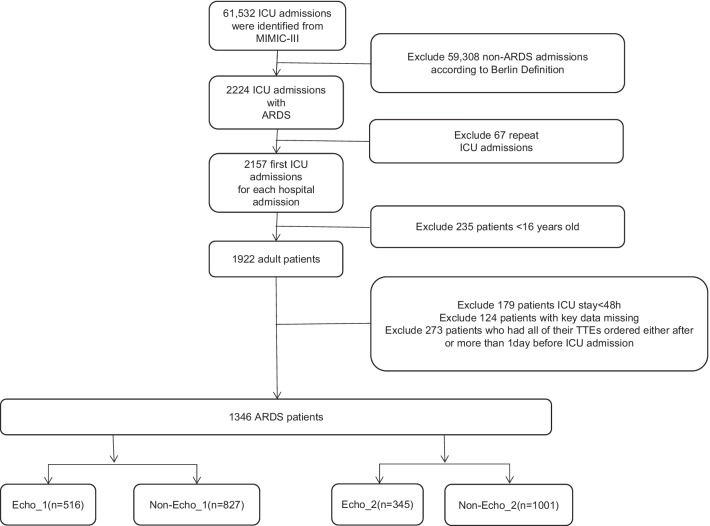
Flow chart of current study

Rather than the assessment of volume and cardiac function in ARDS patients, echocardiography can also be used for the routine assessment of ICU admission. Thus, only the patients who underwent echocardiography within 24 h of mechanical ventilation were enrolled in the study. The enrolled ARDS patients were divided into Echo_1 group and non-Echo_1 group according to whether echocardiography was performed within 24 h of mechanical ventilation. In addition, in order to assess the timing of echocardiography, the patients were also divided into the Echo_2 group and non-Echo_2 group according to whether echocardiography was performed within 24 h after shock (Fig. 1). The variables we extracted or calculated including the baseline characteristics, comorbidities, mean vital signs within 24 h after entering the ICU, and parameters on the first day of mechanical ventilation. The first 24 h after entering the ICU (ie the baseline value) and the extreme values during the ICU stay [ie the maximum and minimum values]. All patients were evaluated for the severity score of organ dysfunction within 24 h after admission.The details of collected data are listed in Additional file 1: Table S1.

The value of lactic acid was not collected in more than 30% of patients in this cohort. Thus, using it directly as a covariate would result in a large number of missing values. Therefore, it was used as a covariate for stratification.

### Outcome indexes

The primary outcome in this study was the 28-d post-admission mortality rate. The secondary outcomes included PAC and PiCCO administration, the ventilator- and vasopressor-free days within 28 d after ICU admission and total intravenous infusion volume on days 1, 2 and 3 of the mechanical ventilation.

### Statistical methods

The data were expressed as mean ± standard deviation, median of interquartile range and ratio (absolute and relative frequency) according to the actual conditions. Continuous data was assessed for normal distribution by Skewness-kurtosis test (sktest) and compared using the Student's *t* test or Mann–Whitney test, while categorical variables were compared using the χ^2^ test or Fisher’s exact test. Statistical analyses were conducted using Stata (version 15.0, StataCorp, College Station, Texas). The propensity score (PS) of patients undergoing TTE was assessed using the psestimate command to minimize the imbalance of variables between the TTE and non-TTE groups [13]. With PS as the weight, the weighted queue was generated by the inverse probability weighting (IPW) model. A *P* < 0.05 was considered to be statistically significant [14].

Multivariate logistic regression was performed on the association between echocardiography and mortality using full models to evaluate the independent effect of echocardiography on the prognosis of patients with ARDS. The final models was built using a stepwise backward elimination with a significance level of 0.05. Potential multicollinearity was tested using a variance inflation factor, with a value of ≥ 5 indicating multicollinearity. Additionally, the log-rank test in the Kaplan-Meter survival analysis was used to compare the different survival rates between each group To exclude the confounding effect of PiCCO and PAC, we performed the sensitivity analysis by excluding patients who receiving either PiCCO or PAC.

### Subgroup analysis

For the Echo_2 group, we performed a propensity score to balance the baseline differences between the two groups, and then performed a χ^2^ test and multivariate logistic regression to clarify the relationship between TTE timing and 28-d mortality.

## Results

Among the 46,476 ICU patients with 61,532 data elements (times of ICU admission) in the MIMIC-III v1.4 database, the Berlin criteria were met in 2,224 elements, and 1,346 patients were finally enrolled in the study and divided into the Echo_1 group: 519 (38.56%) cases, including 219 women (42.2%), and non-Echo_1 group: 827 (61.44%) cases, including 334 women (40.4%). The characteristics of this cohort are shown in Table 1. The simplified acute physiology score(SAPS) and Sequential Organ Failure Assessment(SOFA) scores were higher in the Echo_1 group than those in non-Echo_1 group (SAPS: 44 points *vs*. 40 points, respectively, *P* < 0.001) (SOFA: 7 points *vs*. 6 points, respectively, *P* < 0.001). Besides, the proportions of patients receiving mechanical ventilation (MV) and using vasoactive drugs (VD) were significantly higher in Echo_1 group than those in non-Echo_1 group (MV: 89.4% *vs*. 84.0% respectively) (VD: 51.6% *vs*. 42.8% respectively). It can be seen that the patients undergoing TTE have severer disease than those without TTE (see Table 1 for details). Before PS matching, there were differences in the following parameters between the Echo_1 and non-Echo_1 groups: oxygenation index, positive end expiratory pressure(PEEP), systolic blood pressure, mean arterial pressure(MAP), plateau pressure, Ca^2+^, pH, age, respiratory rate, ARDS severity, ICU type, admission type and the presence or absence of sepsis, SOFA scores, SAPS II score, Oxford Acute Severity of Illness Score (OASIS) and Elixhauser comorbidity score [15–18] (Table 1). After PS matching (1:1) between 440 patients undergoing echocardiography and 440 patients without echocardiography, the imbalance between the Echo_1 and non-Echo_1 groups was significantly reduced (Additional file 2: Fig. S1), and all baseline variables were comparable between the two groups (Table 1). After matching, the 28-d mortality rate significantly declined in the Echo_1 group compared with the non-Echo_1 group (25.9% *vs*. 35.2%, respectively, *P* = 0.003). The infusion volume in the TTE group was significantly smaller than that in non-TTE group on day 1 (6.1 L *vs*. 5.5 L, respectively, *P* = 0.028) and day 3 (3.2 L *vs*. 2.7 L, respectively, *P* = 0.008). The TTE group received more PAC(2% *vs.* 10%, *P* < 0.001), whereas there were no differences on the use of PiCCO between the two groups. The time of using vasopressor and ventilator was significantly shorter in the TTE group than in the non-TTE group (vasopressor-free days within 28 d: 26.8 d *vs*. 27.3 d, respectively, *P* = 0.033, ventilator-free days within 28 d: 17 d *vs*. 19.8 d, respectively, *P* = 0.0202). No statistically significant differences were observed in other secondary endpoints between the two groups (Table 2).

**Table 1:** Baseline characteristics of Echo_1 before and after propensity-score matching

Characteristic	Before matching			After matching		
	Without Echo_1 (*n* = 827)	Echo_1 (*n* = 516)	*P* value	Without Echo_1 (*n* = 440)	Echo_1 (*n* = 440)	*P* value
PaO2/FiO2 ratio(mmHg)	139.5 ± 63.3	128.1 ± 62.8	0.001	130.7 ± 61.7	129.5 ± 62.2	0.779
PEEP( cmH2O)	7.7 ± 3.7	8.1 ± 3.9	0.024	8.1 ± 4.0	8.0 ± 3.8	0.690
Systolic blood pressure (mmHg)	116.2 ± 15.5	113.3 ± 14.8	< 0.001	114.5 ± 15.2	114.1 ± 15.1	0.676
Diastolic blood pressure (mmHg)	60.0 ± 10.2	58.9 ± 9.1	0.050	59.1 ± 10.1	59.4 ± 9.3	0.647
MAP(mmHg)	77.7 ± 10.3	76.3 ± 9.6	0.009	76.6 ± 10.2	76.8 ± 9.7	0.797
Temperature(℃)	37.1 ± 0.7	37.1 ± 0.7	0.782	37.1 ± 0.8	37.1 ± 0.7	0.523
SPO2	96.8 ± 2.9	96.4 ± 3.7	0.049	96.7 ± 3.4	96.4 ± 3.8	0.355
Plateau pressure (cmH2O)	25.8 ± 6.8	26.9 ± 6.9	0.006	26.8 ± 7.0	26.6 ± 6.8	0.624
Tidal volume (ml/kg PBW)	673.7 ± 353.3	652.4 ± 159.5	0.197	647.1 ± 132.4	656.1 ± 164.0	0.371
Peak inspiratory pressure (cmH2O)	32.4 ± 8.3	33.2 ± 7.9	0.060	33.5 ± 8.5	33.0 ± 7.8	0.312
Respiratory rate set	29.7 ± 8.4	31.3 ± 9.0	0.002	30.8 ± 9.2	30.9 ± 8.9	0.861
Blood urea nitrogen (mg/dL)	32.4 ± 25.3	34.5 ± 26.0	0.140	33.5 ± 25.9	33.5 ± 25.4	0.984
calcium (mmol/L)	5.5 ± 3.3	6.1 ± 3.0	< 0.001	6.0 ± 3.2	6.0 ± 3.1	0.784
PaCO2(mm Hg)	43.9 ± 14.0	43.5 ± 13.1	0.628	43.6 ± 13.3	43.5 ± 13.2	0.968
Arterial pH	7.3 ± 0.1	7.3 ± 0.1	0.01	7.3 ± 0.1	7.3 ± 0.1	0.8423
Platelet (× 109 /L)	213.4 ± 130.4	211.1 ± 121.4	0.750	204.2 ± 129.3	209.4 ± 118.1	0.534
Potassium (mmol/L)	4.2 ± 0.88	4.2 ± 0.88	0.757	4.2 ± 0.86	4.2 ± 0.87	0.813
Creatinine (μmol/L)	1.5 ± 1.4	1.5 ± 1.3	0.578	1.4 ± 1.2	1.4 ± 1.2	0.847
Sodium (mmol/L)	138.7 ± 5.3	138.7 ± 5.6	0.804	139.0 ± 5.0	138.6 ± 5.7	0.212
Age	67.9 ± 46.0	62.2 ± 31.0	0.013	63.5 ± 35.0	62.5 ± 33.0	0.672
Weight (kg)	83.7 ± 19.5	87.0 ± 52.3	0.101	84.4 ± 19.2	86.6 ± 54.9	0.426
Minute ventilation (l/min)	12.9 ± 8.8	13.2 ± 5.7	0.460	13.0 ± 4.8	13.0 ± 5.8	0.997
SOFA	6 (4–8)	7 (5–10)	< 0.001	7 (5–9.5)	7 (5–9.5)	0.768
SAPS II	40 (32–50)	44 (34–54)	< 0.001	41 (33–52.5)	43 (33–53)	0.246
OASIS	37 (31–43)	39 (34–45)	< 0.001	39 (33–43)	38.5 (33–44)	0.875
Elixhauser comorbidity score	1 (1–12)	6 (2–13)	0.781	7 (2–12)	6 (2–12.5)	0.573
Heart rate (bpm)	91 (80–103)	92 (80–104)	0.755	91 (80–102)	92 (80–104)	0.770
Mean respiratory rate (/min)	20 (17–24)	21 (18–25)	0.002	21 (17–24)	21 (18–24)	0.83
ARDS severity			0.004			0.971
1	154 (18.6%)	77 (14.8%)		64 (14.5%)	66 (15%)	
2	396 (47.9%)	223 (43.0%)		196 (44.5%)	197 (44.8%)	
3	277 (33.5%)	219 (42.2%)		180 (40.9%)	177 (40.2%)	
ICU type			0.024			0.288
CCU	93 (11.2%)	80 (15.4%)		54 (12.3%)	67 (15.2%)	
CSRU	140 (16.9%)	40 (7.7%)		50 (11.4%)	37 (8.4%)	
MICU	362 ( 43.8%)	265( 51.1%)		209(47.5%)	224(50.9%)	
SICU	111( 13.4%)	67 (12.9%)		61(13.9%)	57(13.0%)	
TSICU	121 (14.6%)	67 (12.9%)		66 (15%)	55 (12.5%)	
Admission type			0.001			0.068
ELECTIVE	102 (12.3%)	33 (6.4%)		38 (8.6%)	6.6 (29)	
EMERGENCY	690 (83.4%)	468 (90.2%)		376 (85.5%)	90.2 (397)	
URGENT	35 (4.2%)	18 (3.5%)		26 (5.9%)	14 (3.2%)	
Gender			0.511			0.892
Male	493 (59.6%)	300 (57.8%)		253 (57.5%)	251 (57.0%)	
Diabetes	195 (23.6%)	123 (23.7%)	0.960	102 (23.2%)	99 (22.5%)	0.810
Hypertension	261 (31.6%)	144(27.7%)	0.138	127 (28.9%)	124 (28.2%)	0.823
COPD	129(15.4%)	72 (13.9%)	0.387	61 (13.7%)	61 (13.7%)	1.000
Sepsis	563 (68.1%)	392(75.5%)	0.003	316(71.8%)	321(73.0%)	0.706
CHF	306 (37.0%)	204 (39.3%)	0.396	174(39.5%)	174(39.5%)	1.000
AFIB	249 (30.1%)	135 (26.0%)	0.105	118(26.8%)	112(25.5%)	0.645
Renal	76 (9.2%)	44 (8.5%)	0.655	38 (8.6%)	36 (8.2%)	0.808
Liver	62 (7.5%)	43 (8.3%)	0.600	40 (9.1%)	35 (8.0%)	0.546
CAD	164 (19.8%)	85 (16.4%)	0.112	77 (17.5)	76 (17.3%))	0.929
Stroke	70 (8.5%)	42(8.1%)	0.810	33(7.5%)	34(7.7%)	0.899
Malignancy	135 (16.3%)	90(17.3%)	0.626	72(16.4%)	77(18.0%)	0.531
Day of ICU admission			0.406			0.296
Monday	140 (16.9%)	78 (15.0%)		47 (10.7%)	62 (14.1%)	
Tuesday	109 (13.2%)	66 (12.7%)		88 (20%)	69 (15.7%)	
Wednesday	128 (15.5%)	89 (17.1%)		59 (13.4%)	58 (13.2%)	
Thursday	114 (13.8%)	62 (11.9%)		63 (14.3%)	80 (18.2%)	
Friday	140 (16.9%)	79 (15.2%)		50 (11.4%)	52 (11.8%)	
Saturday	98 (11.9%)	64 (12.3%)		76 (17.3%)	66 (15%)	
Sunday	98 (11.9%)	81 (15.6%)		57 (13.0%)	53 (12.0%)	
Lactic acid			0.119			0.395
Missing value	341(41.2%)	229(44.1%)		207 (47.0%)	190 (43.2%)	
< 4	82 (18.6%)	64 (12.3%)		40 (10.2%)	55 (12.5%)	
> 4	404 (48.9%)	206 (39.7%)		188 (42.7%)	195 (44.3%))	
Vasopressin use (*n*, %)	354 (42.8%)	268 (51.6)	< 0.001	206 (46.8%)	211 (47.9%)	0.736

*PO*_*2*_ oxygen partial pressure, *FiO2* Fraction of inspiration O2, *PEEP* positive end expiratory pressure, *MAP* Mean arterial pressure, *SpO*_*2*_ pulse oxygen saturation, *PBW* Parts by Weight, *PCO*_*2*_ carbon dioxide partial pressure, *SAPS* simplified acute physiology score, *SOFA* sequential organ failure assessment score, *OASIS* Oxford acute severity of illness score, *BUN* blood urea nitrogen, *CCU* Coronary Care Unit, *CSRU* Cardiovascular Surgery Rehabilitation Unit, *MICU* Medical Intensive Care Unit, *SICU* Surgical Intensive Care Unit, *TSICU* Trauma Surgery Intensive Care Unit, *CHF* Congestive heart failure, *AFIB* atrial fibrillation, *COPD* chronic obstructive pulmonary disease, *CAD* coronary artery disease

**Table 2:** Outcomes of patients in matched cohort of Echo_1

	No Echo_1(*n* = 440)	Echo_1(*n* = 440)	*P* value
*Primary outcome*			
28-day mortality (*n*, %)	155 (35.2%)	114 (25.9%)	0.003
*Secondary outcomes*			
PAC (*n*, %)	9 (2%)	44 (10%)	< 0.001
PiCCO (*n*, %)	30 (6.8%)	45 (10.2%)	0.07
ICU mortality (*n*, %)	82 (18.6%)	76 (17.3%)	0.598
Length of ICU stay(d)	11 (5–19)	10 (5–15)	0.140
Length of hospital stay(d)	18 (10–28)	14 (9–23)	< 0.001
IV fluid day 1 (mL)	6072 (3534–10,235)	5459 (3017–8903)	0.028
IV fluid day 2 (mL)	3829(2036–6926)	3454.877 (2096 -6149.517)	0.098
IV fluid day 3 (mL)	3176 (1653 -6021)	2666 (1326 -5070)	0.008
Ventilation free days in 28 days	17.0 (0–24.7)	19.8 (0–25.2)	0.020
Vasopressor free days in 28 days	26.8 (0–28)	27.3 (0–28)	0.033

*PAC* pulmonary artery catheter; *PiCCO* pulse index continuous cardiac output

The univariate logistic regression analysis for 28-d mortality was shown in Additional File 3: Table S2. There was a significant association between patients’ variables (Blood urea nitrogen, Arterial pH.

Plateau pressure, SOFA score, Malignancy, Temperature, Mean respiratory rate, Systolic blood pressure, Age) and 28-d mortality. In multivariate logistic regression analysis, after adjusting for the listed clinical confounders, we found that TTE patients had a significantly lower 28-day mortality risk than non-TTE patients (OR 0.71, 95% CI 0.55–0.91, *P* = 0.007) (Table 3).

**Table 3:** Adjust ORs using Echo_1 and Echo_2 as the design variable in patients with ARDS

Model1				Model 2			
variable	OR	95%CI	*P*	variable	OR	95%CI	*P*
Echo_1	0.70	0.55–0.91	0.007	Echo-2	1.02	0.79–1.34	0.844
Blood urea nitrogen	1.00	1.00–1.01	0.002	Age	1.00	1.00–1.00	0.026
Age	1.00	1.00–1.00	0.020	Arterial pH	0.19	0.04–0.87	0.032
Plateau pressure	1.02	1.00–1.04	0.005	Temperature	0.65	0.52–0.81	< 0.001
Systolic blood pressure	0.99	0.98–0.99	0.048	CHF	1.40	1.01–1.96	0.038
Malignancy	1.40	1.03–1.92	0.028	Malignancy	1.53	1.03–2.27	0.034
Temperature	0.70	0.59–0.84	< 0.001	Plateau pressure	1.03	1.01–1.06	0.003
Mean respiratory rate	1.03	1.00–1.05	0.017	Hypertension	0.59	0.41–0.84	0.004

Model1 contains 519 patients with TTE in Echo_1

Model2 contains 345 patients with TTE in Echo_2

*CHF* Congestive heart failure

**Fig. 2:**
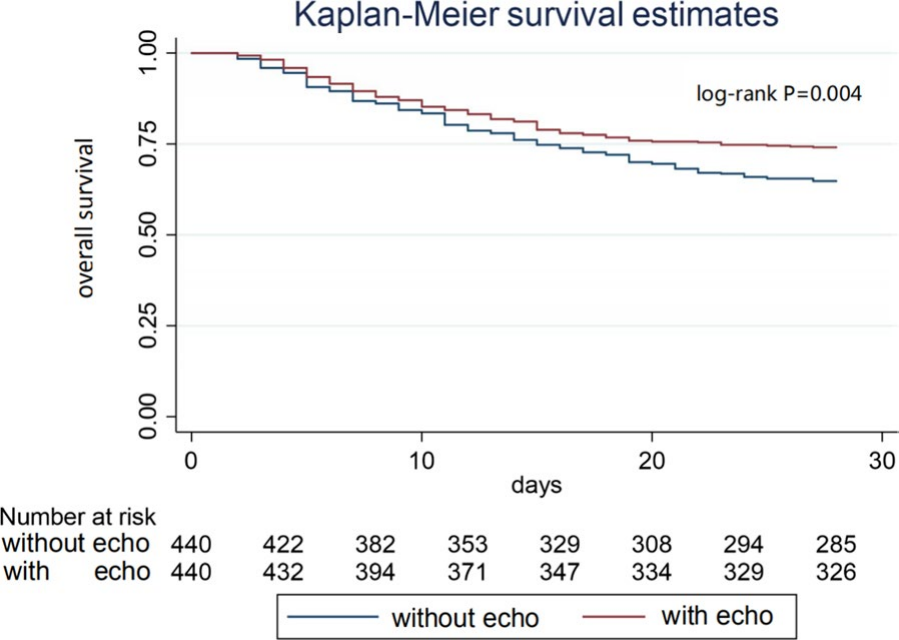
Kaplan–Meier analysis of survival time in the Echo_1 group *vs.* the without Echo_1 group

Kaplan–Meier curves showed that TTE was strongly associated with improved survival (*P* = 0.004 by log-rank test, Fig. 2).

### Subgroup analysis

Before PS matching, there were differences between the Echo_2 group and non-Echo_2 group in the following parameters: the oxygenation index, PEEP, systolic blood pressure, MAP, plateau pressure, peak airway pressure, blood urea nitrogen(BUN), weight, minute volume, pH, SOFA score, SAPSII, OASIS, respiratory rate, ARDS severity, ICU type, lactic acid and the presence or absence of sepsis (Additional file 4: Table S3). After PS matching (1:1) between 314 patients undergoing echocardiography and 316 patients without echocardiography, the imbalance between the Echo_2 and non-Echo_2 groups was significantly reduced and all baseline variables were comparable between the two groups (Additional file 5: Fig S2). After matching, there was no significant difference in the 28-d mortality rate between the Echo_2 and non-Echo_2 groups (32.9% *vs*. 29.9%, respectively, *P* = 0.418). The analysis results of secondary endpoints revealed a significantly shorter length of stay in the TTE group than that in the non-TTE group (12.5 d *vs*. 11 d, respectively, *P* = 0.017), while other secondary endpoints showed no statistically significant differences between the two groups (Table 4) (Additional file 5: Fig. S2).

**Table 4:** Outcomes of patients in matched cohort of Echo_2

	No Echo_2 (*n* = 316)	Echo_2 (*n* = 314)	*P* value
*Primary outcome*			
28-day mortality (*n*, %)	104 (32.9%)	97 (29.9%)	0.418
*Secondary outcomes*			
PAC (*n*, %)	23 (7.3%)	30 (9.5%)	0.03
PiCCO (*n*, %)	25 (7.9%)	50 (15.9%)	0.54
ICU mortality (*n*, %)	67 (21.2%)	58 (17.9%)	0.292
Length of ICU stay (d)	11 (5–18)	12.5 (7–21)	0.017
Length of hospital stay(d)	17 (9–25.5)	18 (10–29)	0.275
IV fluid day 1 (mL)	6884 (4045–11061)	6945 (4232–11044)	0.950
IV fluid day 2 (mL)	4078 (2308–6906)	4455 (2586–6998)	0.470
IV fluid day 3(mL)	3606 (1887–6960)	2910 (1410 -5459)	0.442
Ventilation free days in 28 days	20 (0–26)	19 (0–25)	0.111
Vasopressor free days in 28 days	27.5 (0–28)	26.4 (0–27.7)	< 0.001

In multivariable logistic regression analysis, after adjusting for the clinical confounders listed, we found that TTE patients compared with non-TTE patients had not association with the 28-d mortality (OR 1.02, 95%CI 0.79–1.34, *P* = 0.844)(Table 3).

### Senitivity analysis

**Fig. 3:**
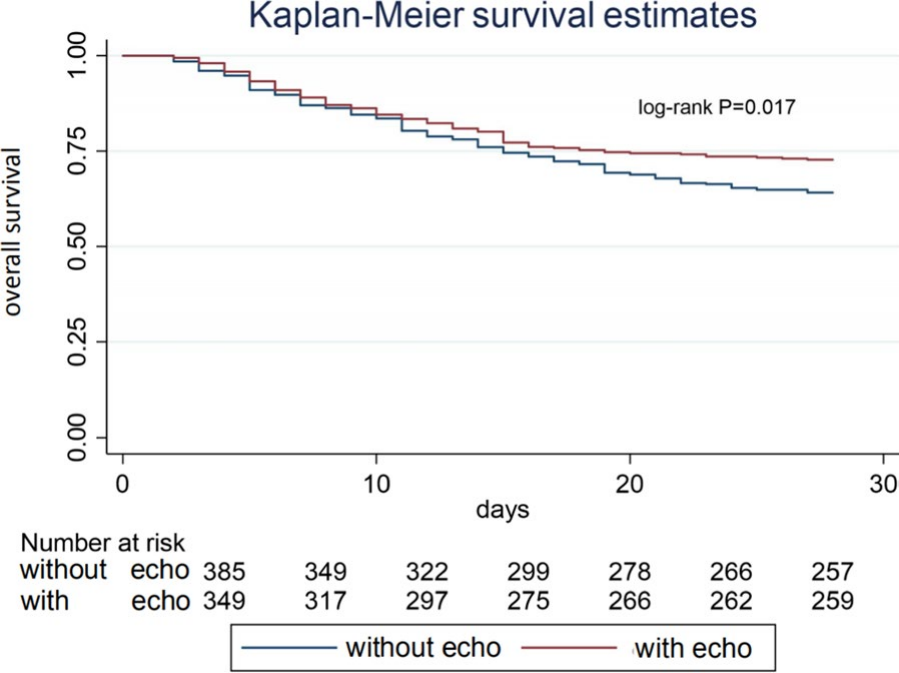
Kaplan–Meier analysis of survival time after excluding patients received with PAC or PiCCO

To illuminate the impact of echocardiography on the outcomes of ARDS patients, we performed a sensitivity analysis after excluding patients received with PAC or PiCCO. There was a significant difference in the 28-d mortality rate between the TTE and non-TTE groups (27.2% *vs*. 35.9%, respectively, *P* = 0.011). Kaplan–Meier curves showed that TTE was strongly associated with improved survival (*P* = 0.017 by log-rank test, Fig. 3).

## Discussion

ARDS is a life-threatening pulmonary disease with a poor prognosis and an increased mortality rate [19, 20]. Such an adverse outcome may result from circulatory failure rather than hypoxemia [2]. Therefore, hemodynamic monitoring plays a very important role in the management of ARDS patients. Clinically widely used hemodynamic monitoring strategies, including CVP, PAC, and PiCCO. However, when CVP is within a relatively normal range, the ability of prediction to guide fluid management is limited [21]. Therefore, it is not reasonable to use CVP alone to monitor hemodynamics [22] As for PAC, previous studies have shown no significant improvement on mortality [23–25] and even more complications than central venous catheter guided therapy [26]. The risk of the PiCCO cannulation limits its use in critically ill or high-risk patients with complex and severe hemodynamic impared [27] Furthermore, it does not improve patient outcomes compared with CVP [6], 28] This is because the decisive question is how clinicians use the information obtained for subsequent management. Critical ultrasound examination is a rapid, non-invasive and reproducible operation, with a dynamic and visual presentation of the results, combining the monitoring results and diagnosis and treatment thoughts for critical disease. It plays an increasingly important role in the clinical diagnosis and treatment of ARDS.

Previous studies mainly focused on its diagnosis and treatment of ARDS, while the influencing factors for its prognosis and the value of ultrasonography for the prognostic evaluation have rarely been studied. It is of great significance to determine the actual application value of the auxiliary examination for the patients. First, there should be good reasoning for the patients requiring examination. Second, unnecessary examination and occupation of the medical resources should be avoided to ease the economic burden on the individuals, society and country. Therefore, continuous testing in clinical practice is needed for any new technique to determine its application value. The MIMIC-III database, established based on the electronic medical record, is a continuously updated dynamic data system, which reflects the diagnosis and treatment process of critically ill patients and has been commonly used by the researchers of intensive care medicine [29].To the best of our knowledge, this is the first study assessing the effect of echocardiography on the prognosis of ARDS patients. In this work, patients undergoing TTE had a higher disease severity score and more comorbid conditions, suggesting a severer disease degree. It was found that the 28-d mortality rate of patients undergoing TTE significantly declined after adjusting the confounding factors. Several hypotheses were proposed to explain the survival benefit, and some variables were compared between the TTE and non-TTE groups. The infusion volume in the TTE group was smaller at day 1 and day 3 after ventilation. Vasopressors were used more often in the TTE group, probably because TTE promotes the timely use of vasopressors. Patients in the TTE group also stopped taking vasopressors earlier than those in non-TTE group, which could be related to several factors according to the analysis of PS matching. First, studies have shown that acute pulmonary heart disease occurs in 20–25% of ARDS patients [30], and many obstructive factors, including hypoxia-induced pulmonary vasospasm, hypercapnia, high airway pressure, inflammatory factor-induced vasoconstriction and lung volume collapse cause an increased pulmonary vascular resistance, which greatly affects the right heart function and pulmonary circulatory resistance [31]. During positive pressure ventilation, pulmonary vessels are compressed by the stretched alveoli, which leads to increased pulmonary artery resistance and obviously reduces the pulmonary circulation blood flow [32]. The increase in PEEP raises the pulmonary vascular resistance, which leads to right heart dysfunction and may eventually result in the occurrence and development of shock. Echocardiography assessment of the right heart function during treatment can reveal the major cause of hemodynamic involvement or instability, because the status of the right heart involvement greatly varies among different types of shock, which directly affects the development and implementation of the clinical therapeutic regimen. Second, in terms of fluid management, right heart enlargement can be caused by an acute increase in the blood volume. Acute right heart enlargement occurs when there is a failure of compensatory fluid discharge due to renal insufficiency or low MAP. This results in left ventricular diastolic restriction through the ventricular septum and the pericardium, increasing the left ventricular filling pressure, and thus the extravascular lung water [33].The fluid management strategy test of the ARDS Collaboration in 2006 well established that patients with ARDS can mostly benefit from the conservative fluid management strategy through shock correction (vasopressor dependence), keeping the circulatory stability and guaranteeing organ perfusion. Although the conservative strategy does not reduce the 60-d mortality, it can shorten the duration of mechanical ventilation and length of ICU stay and ameliorate oxygenation without increasing the incidence of other organ dysfunction [34]. Moreover, a systematic review and meta-analysis covering 2051 patients with sepsis and/or ARDS in 11 randomized trials in 2017 found no significant difference in the mortality rate between the restricted fluid management group and routine treatment group [35]. However, the ventilator-free duration was found to be significantly increased and the length of ICU stay was significantly shortened in the restricted fluid management group. Our study showed that the infusion volume was smaller in the TTE group, which may have contributed to the improved survival. Third, vasoactive drugs are an important treatment means to lower the pulmonary circulatory pressure. As the distribution of related receptors varies, attention should be paid to the different effects of vasoactive drugs on pulmonary circulation and systemic circulation. Generally, vasodilators may also affect the systemic circulation when they act on the pulmonary circulation, a contradiction that may be sharper in severely ill patients. On the one hand, vasodilators expand the pulmonary artery and lower the pulmonary circulatory resistance, which supports the restoration of the right heart function and reduces the central venous pressure (CVP). On the other hand, vasodilators reduce the systemic circulation pressure, leading to circulatory instability. In particular, when less obvious decline in PAP and obvious decline in the systemic circulation pressure are observed when vasodilators are used, the transseptal pressure will be altered, which results in a leftward shift of the interventricular septum, a significant decrease in the left ventricular end-diastolic volume and a decline in the cardiac output. As a result, the systemic circulation pressure is further decreased, leading to an autonomous vicious cycle of the right heart [36]. It has been confirmed that these drugs can be applied under the guidance of tricuspid annular plane systolic excursion(TAPSE), right/left ventricular area ratio and eccentricity index [30]. The above-mentioned discussion may include the reasons for the improved mortality of ARDS patients by ultrasonography.

In addition, the patients were divided into the Echo_2 group and non-Echo_2 group according to the time of ultrasonography. Unlike the Echo_1 group, patients in the Echo_2 group showed no improvement regarding the clinical outcome, with no significant difference in the 28-d mortality between the Echo_2 group and control group. It is believed that ultrasonography should be performed as soon as possible on ARDS patients to reduce the mortality rate to the largest extent. However, no relevant studies have been presented. Hence, larger-scale prospective randomized controlled trials should be performed in the future to determine the timing of ultrasonography for ARDS patients. Although early assessment will not necessarily help to avoid further lung injury throughout the course of the disease, it can enable us to adjust the ventilation strategy, thereby ameliorating the prognosis. Based on our experience, we can suggest that ALI or ARDS patients receive ultrasonography at the time of ICU admission, and regular review should be performed according to the disease condition. Echocardiography at admission can provide valuable information, not only about the current clinical conditions, but also about preexisting diseases (*e.g*., severe right ventricular hypertrophy suggests the presence of chronic lung disease). If the condition is stable, then echocardiography should be performed at least once a week before weaning and after extubation (*i.e*., fluid therapy monitoring). Moreover, echocardiographic can assist in determining the cause (or auxiliary factor) of the progressive respiratory failure, such as systolic pulmonary artery pressure(sPAP) elevation, right ventricular dilatation, progressive right or left ventricular failure or individualized treatment. Re-examination (ventilation and non-ventilation) should be performed whenever right ventricular dilation or dysfunction is developed (even by conventional echocardiography), other options (*i.e*., inhalation of nitric oxide and prone position) should also be considered, and patients should be monitored more closely using echocardiography.

Our study had some limitations. First, despite the large sample size of real data, the data came from a single medical center, and there may be deviations among the subjects in the medical level, habits and population. Second, this was a retrospective analysis, so a large amount of data might have been eliminated, and there might have been a selection bias due to the lack of key information and other reasons during data extraction. Although TTE is a non-invasive and convenient operation, it has a poor repeatability. We could not assess the consistency of TTE in this study, resulting in a measurement bias. Therefore, we suggest designing a prospective multi-center study based on similar studies to further observe the effects of TTE on patients with severe ARDS. Third, although the 28-d mortality rate was explored, some significant outcome variables were not considered in the analysis, including long-term mortality and ICU readmission. Finally, the MIMIC-III database included the cases before 2012. Some studies suggest expanding the Berlin criteria for ARDS to include patients who have undergone high-flow nasal oxygen therapy (at least 30 L/min) and meet other standards in the Berlin criteria [37], which may lead to a result deviation in this study.

## Conclusion

TTE was associated with improved 28-day outcomes of critically ill ARDS patients. Its mechanism remains to be explored, but under the guidance of the TTE results, we suggest that it may be related to the evaluation of right heart function and adjustment of fluid and vasoactive drugs. In future work, more large-scale prospective studies are needed to explore the influencing mechanism of ultrasound to the long-term prognosis of patients with ARDS and the timing of ultrasound use.

## Supplementary Information

**Additional file 1.** Table S1.

**Additional file 2.** Figure S1.

**Additional file 3.** Table S2.

**Additional file 4.** Table S3.

**Additional file 5.** Figure S2.

## Data Availability

The datasets analysed during the current study are available in the MIMIC-III repository, https://physionet.org/content/mimiciv/0.4/.

## Abbreviations

MIMIC-III: The medical information mart for intensive care III
ARDS: Acute respiratory distress syndrome
TTE: Transthoracic echocardiography
ICU: Intensive care unit
PS: Propensity score
OR: Odds ratio
PAP: Pulmonary arterial pressure
RV: Right ventricle
CT: Computer tomography
APACHEII: Acute physiology and chronic health evaluation II
MIT: Massachusetts institute of technology
IPW: Inverse probability weighting
SAPS: Simplified acute physiology score
SOFA: Sequential organ failure assessment
MV: Mechanical ventilation
VD: Vasoactive drugs
PEEP: Positive end expiratory pressure
MAP: Mean arterial pressure
OASIS: Oxford acute severity of illness score
BUN: Blood urea nitrogen
CVP: Central venous pressure
TAPSE: Tricuspid annular plane systolic excursion
sPAP: Systolic pulmonary artery pressure
CHF: Congestive heart failure
AFIB: Atrial fibrillation
CKD: Chronic kidney disease
COPD: Chronic obstructive pulmonary disease
CAD: Coronary artery disease
SpO2: Pulse oxygen saturation
PO2: Oxygen partial pressure
PCO2: Carbon dioxide partial pressure
PAC: Pulmonary artery catheter
PiCCO: Pulse index continuous cardiac output
