# Supplementary material for: Effects of transthoracic echocardiography on the prognosis of patients with acute respiratory distress syndrome: a propensity score matched analysis of the MIMIC-III database
Source: BMC Pulm Med. 2022 Jun 25;22:247. doi: 10.1186/s12890-022-02028-5 (PMC9233371; doi:10.1186/s12890-022-02028-5)
Supplement: Supplementary file 1 — Additional file 1. Table S1. [file 12890_2022_2028_MOESM1_ESM.docx]

| Covariates | Demographic data and information at admission: age, gender, weight, day of the week of admission, admission time and severity of disease at admission assessed by SAPS, SOFA score, OASIS and Elixhauser comorbidity score |
| --- | --- |
| Comorbidities | CHF, AFIB, chronic kidney disease, liver disease, COPD, CAD, stroke, malignancy, diabetes, hypertension and sepsis. All comorbidities were identified by the ICD-9 code recorded |
| Vital signs | Mean MAP, mean systolic blood pressure, mean diastolic blood pressure, mean respiratory rate, mean heart rate, mean body temperature (F) and SpO_2_ at admission |
| Intervention measures | Use of mechanical ventilation and vasopressors after admission |
| Laboratory results | Platelet count, sodium, potassium, calcium, lactic acid, creatinine, BUN, pH, PO_2_, PCO_2_ |
| Mechanical ventilation parameters | Maximum plateau pressure, peak inspiratory pressure, tidal volume, PEEP and respiratory rate |

Table S1 Details of the extracted data

**Abbreviations:**SAPS=simplified acute physiology score, SOFA=sequential organ failure assessment score, OASIS=Oxford acute severity of illness score CHF=Congestive heart failure , AFIB=atrial fibrillation, COPD=chronic obstructive pulmonary disease ,CAD= coronary artery disease,MAP=Mean arterial pressure, SpO_2_=pulse oxygen saturation BUN=blood urea nitrogen, PO_2=_oxygen partial pressure, PCO_2=_carbon dioxide partial pressure, PEEP=positive end expiratory pressure
