# Supplementary figures and images for: Effects of transthoracic echocardiography on the prognosis of patients with acute respiratory distress syndrome: a propensity score matched analysis of the MIMIC-III database
Source: BMC Pulm Med. 2022 Jun 25;22:247. doi: 10.1186/s12890-022-02028-5 (PMC9233371; doi:10.1186/s12890-022-02028-5)

Figure S1 Echo\_1 propensity score matching graph

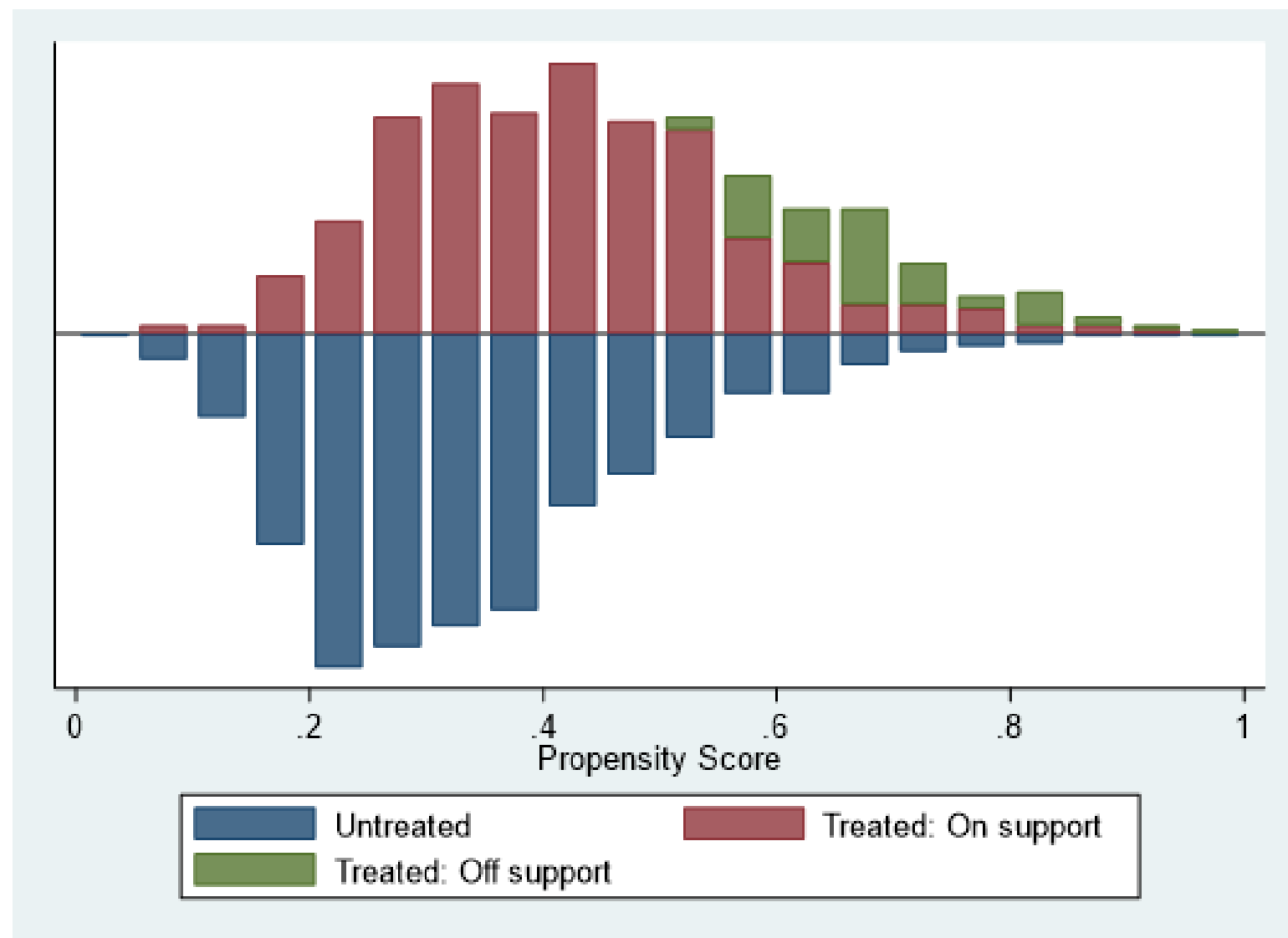

Supplement: Supplementary file 2 — Additional file 2. Figure S1. [file 12890_2022_2028_MOESM2_ESM.pdf]

Figure S2 Echo\_2 propensity score matching graph

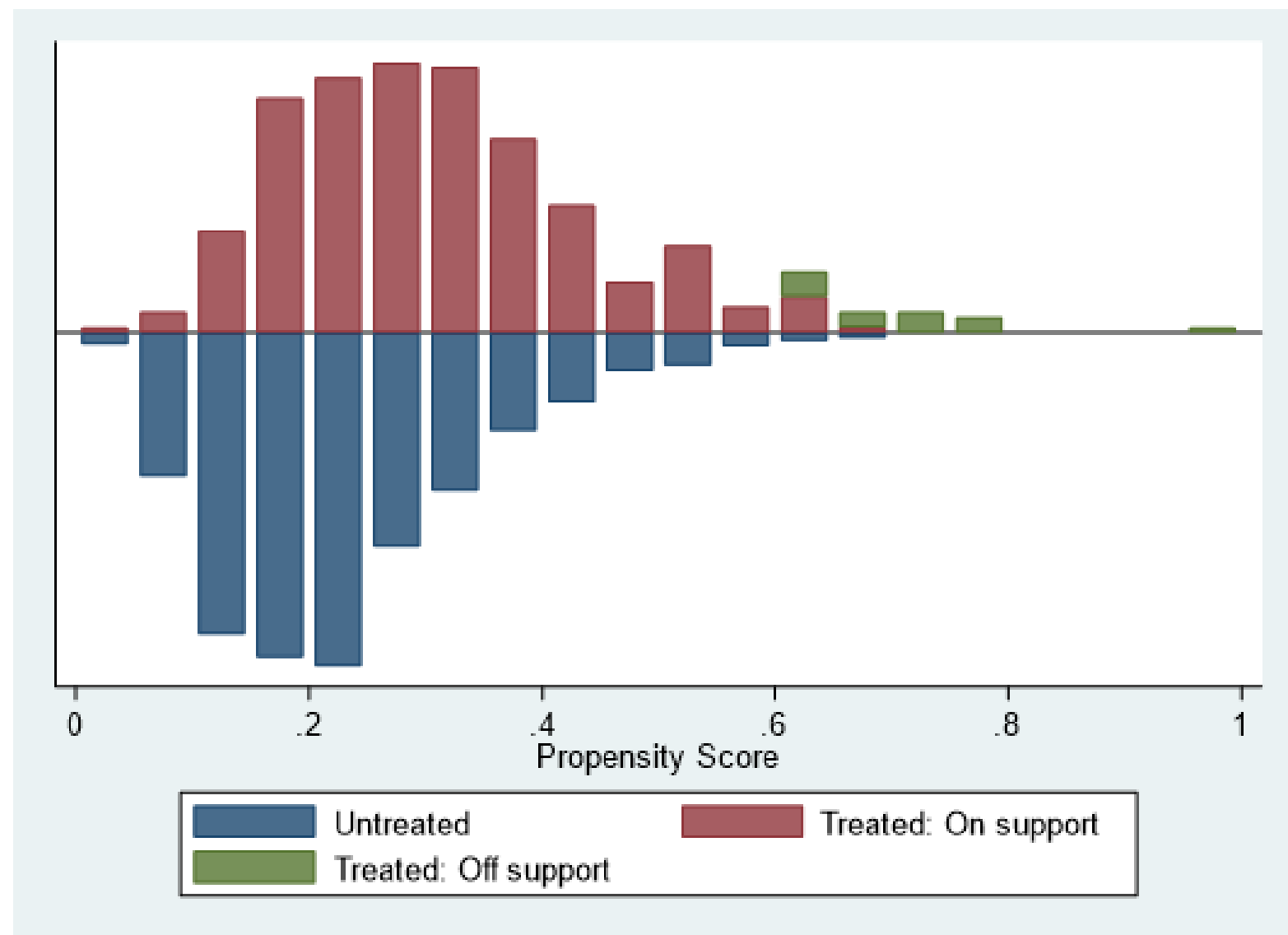

Supplement: Supplementary file 5 — Additional file 5. Figure S2. [file 12890_2022_2028_MOESM5_ESM.pdf]
