# Supplementary material for: Effects of transthoracic echocardiography on the prognosis of patients with acute respiratory distress syndrome: a propensity score matched analysis of the MIMIC-III database
Source: BMC Pulm Med. 2022 Jun 25;22:247. doi: 10.1186/s12890-022-02028-5 (PMC9233371; doi:10.1186/s12890-022-02028-5)
Supplement: Supplementary file 3 — Additional file 3. Table S2. [file 12890_2022_2028_MOESM3_ESM.docx]

Table S2 Univariate logistics regression analysis of all patients on 28-d mortality

| Variables | 28-d mortality  OR 95% CI p-value |
| --- | --- |
| Echo_1 | 0.78(0.61,0.99) 0.041 |
| Echo_2 | 1.02(0.78,1.34) 0.844 |
| PaO2/FiO2 ratio(mmHg) | 0.99(0.99,1.00) 0.209 |
| PEEP( cmH2O) | 0.99(0.96,1.02) 0.731 |
| Systolic blood pressure (mmHg) | 0.99(0.98,0.99) 0.023 |
| Temperature(℃) | 0.69(0.59,0.82) <0.001 |
| SPO2 | 0.97(0.94,1.01) 0.203 |
| Plateau pressure (cmH2O) | 1.02(1.00,1.04) 0.007 |
| Tidal volume (ml/kg PBW) | 0.99(0.99,1.00) 0.266 |
| Peak inspiratory pressure (cmH2O) | 1.01(0.99,1.02) 0.075 |
| Blood urea nitrogen (mg/dL) | 1.01(1.0-1.01) <0.001 |
| Calcium (mmol/L） | 1.02(0.98,1.06) 0.235 |
| Arterial pH | 0.32(0.11,0.93) 0.038 |
| Platelet (×109 /L） | 0.99(0.99-1.00) 0.773 |
| Potassium (mmol/L） | 1.03(0.91-1.18) 0.562 |
| Sodium (mmol/L） | 0.99(0.97-1.02) 0.621 |
| Age | 1.00(1.00,1.00) 0.002 |
| Weight (kg) | 0.99(0.99,1.00) 0.229 |
| Minute ventilation (l/min) | 0.99(0.98-1.01) 0.744 |
| SOFA | 1.06(1.02,1.09) <0.001 |
| Heart rate (bpm) | 0.99(0.99,1.00) 0.865 |
| Mean respiratory rate (/min) | 1.02(1.00,1.05) 0.016 |
| ARDS severity | 1.15(0.97,1.36) 0.086 |
| 1 |  |
| 2 |  |
| 3 |  |
| ICU type | 0.94 (0.85,1.04) 0.229 |
| CCU |  |
| CSRU |  |
| MICU |  |
| SICU |  |
| TSICU |  |
| Admission type | 0.94 (0.69,1.29) 0.700 |
| ELECTIVE |  |
| EMERGENCY |  |
| URGENT |  |
| Gender | 0.82(0.65,1.04) 0.116 |
| Male |  |
| Diabetes | 0.86(0.65,1.13) 0.297 |
| Hypertension | 0.77(0.60,1.00) 0.059 |
| COPD | 0.88(0.63,1.23) 0.470 |
| Sepsis | 1.15(0.89,1.50) 0.274 |
| CHF | 1.17(0.92,1.49) 0.181 |
| AFIB | 1.03(0.79,1.33) 0.818 |
| Renal | 1.39(0.94,2.05) 0.097 |
| Liver | 1.23(0.81,1.88) 0.321 |
| CAD | 0.89(0.65,1.21) 0.468 |
| Stroke | 0.84(0.54,1.30) 0.437 |
| Malignancy | 1.52(1.13,2.05) 0.006 |
| Day of ICU admission | 0.93(0.99,1.05) 0.841 |
| Monday |  |
| Tuesday |  |
| Wednesday |  |
| Thursday |  |
| Friday |  |
| Saturday |  |
| Sunday |  |
| Lactic acid | 1.07(0.94,1.21) 0.257 |
| Vasopressin use | 0.93(0.74,1.18) 0.576 |

**Abbreviations:**PO_2_=oxygen partial pressure,FiO2=Fraction of inspiration O2, PEEP=positive end expiratory pressure, SpO_2_=pulse oxygen saturation, PBW=Parts by Weight, SOFA=sequential organ failure assessment score, BUN=blood urea nitrogen, CCU=Coronary Care Unit , CSRU=Cardiovascular Surgery Rehabilitation Unit, MICU=Medical Intensive Care Unit, SICU=Surgical Intensive Care Unit，TSICU=Trauma Surgery Intensive Care Unit, CHF=Congestive heart failure, AFIB=atrial fibrillation, COPD=chronic obstructive pulmonary disease, CAD= coronary artery disease.
