# Supplementary material for: Effects of transthoracic echocardiography on the prognosis of patients with acute respiratory distress syndrome: a propensity score matched analysis of the MIMIC-III database
Source: BMC Pulm Med. 2022 Jun 25;22:247. doi: 10.1186/s12890-022-02028-5 (PMC9233371; doi:10.1186/s12890-022-02028-5)
Supplement: Supplementary file 4 — Additional file 4. Table S3. [file 12890_2022_2028_MOESM4_ESM.docx]

Table S3 Baseline characteristics of Echo_2 before and after propensity-score matching

| Characteristic | Before matching | | | After matching | | |
| --- | --- | --- | --- | --- | --- | --- |
|  | Without Echo_2  (n=1001) | Echo_2  (n=345) | P value | Without Echo_2  (n=316) | Echo_2  (n=314) | P value |
| PaO2/FiO2 ratio(mmHg) | 139.0±63.6 | 124.0± 61.3 | <0.001 | 125.2±58.6 | 125.2±61.9 | 0.768 |
| PEEP( cmH2O) | 7.6±3.6 | 8.5±4.0 | <0.001 | 8.5±4.0 | 8.2±3.9 | 0.946 |
| Systolic blood pressure (mmHg) | 116.5±15.7 | 111.0± 13.3 | <0.001 | 111.2±12.8 | 111.5±13.1 | 0.780 |
| Diastolic blood pressure (mmHg) | 59.9± 10.2 | 58.8±8.6 | 0.089 | 58.5±9.3 | 58.9±8.6 | 0.494 |
| MAP(mmHg) | 77.7±10.4 | 75.6±9.0 | <0.001 | 75.4±8.8 | 75.8±9.0 | 0.496 |
| Temperature(℃) | 37.1±0.7 | 37.0±0.7 | 0.105 | 37.1±0.7 | 37.1±0.7 | 0.648 |
| SPO2 | 96.7± 3.3 | 96.7±3.0 | 0.866 | 96.7±2.5 | 96.7±3.0 | 0.807 |
| Plateau pressure (cmH2O) | 25.8±6.8 | 27.5±6.9 | <0.001 | 27.5±7.2 | 27.4±7.1 | 0.744 |
| Tidal volume (ml/kg PBW) | 666.5±282.8 | 662.5±325.4 | 0.060 | 686.0±462.5 | 659.5±164.0 | 0.732 |
| Peak inspiratory pressure (cmH2O) | 32.3±8.2 | 34.0±7.8 | <0.001 | 34.3±8.5 | 33.6±7.8 | 0.837 |
| Respiratory rate | 30.0±8.5 | 31.2±9.1 | 0.020 | 30.6±8.3 | 31.9±9.9 | 0.654 |
| Blood urea nitrogen (mg/dL) | 32.2±24.6 | 36.2± 28.2 | 0.011 | 35.9±27.9 | 35.0±26.3 | 0.632 |
| Calcium (mmol/L） | 5.7±3.3 | 5.8±3.1 | 0.612 | 5.6±3.3 | 6.1±3.0 | 0.781 |
| PaCO2(mm Hg) | 44.0±14.0 | 42.9±12.8 | 0.191 | 43.4±12.6 | 42.4±12.2 | 0.520 |
| Arterial pH | 7.3±0.1 | 7.3±0.1 | <0.001 | 7.3±0.1 | 7.3±0.1 | 0.277 |
| Platelet (×109 /L） | 212.9±126.4 | 211.3±128.9 | 0.845 | 219.9±134.5 | 210.6±122.4 | 0.875 |
| Potassium (mmol/L） | 4.2±0.87 | 4.3±0.90 | 0.363 | 4.2±0 .9 | 4.3±0.9 | 0.596 |
| Creatinine (μmol/L） | 1.5±1.4 | 1.6± 1.3 | 0.245 | 1.4±1.3 | 1.6±1.3 | 0.854 |
| Sodium (mmol/L） | 138.7±5.3 | 138.8± 5.6 | 0.757 | 138.9±5.4 | 138.8±5.6 | 0.955 |
| Age | 66.6± 42.5 | 62.9±36.1 | 0.140 | 65.2±34.9 | 61.4±31.7 | 0.881 |
| Weight (kg) | 83.7±20.9 | 88.5±61.3 | 0.034 | 83.9±22.0 | 85.9±26.0 | 0.564 |
| Minute ventilation (l/min) | 12.8± 7.4 | 13.8±8.7 | 0.032 | 14.1±10.1 | 13.4±5.3 | 0.876 |
| SOFA | 6（4-8） | 8（5-10） | <0.001 | 7.5(5-10) | 8(5-10) | 0.768 |
| SAPS II | 40(32-50) | 45（35-56） | <0.001 | 44(35-55) | 44(34.5-55) | 0.908 |
| OASIS | 37（32-43） | 40（34-46） | <0.001 | 39(34-46) | 40(33-45) | 0.572 |
| Elixhauser comorbidity score | 6（1-12） | 7（2-13） | 0.258 | 7(3-12.5) | 7(2-13) | 0.801 |
| Heart rate (bpm) | 91(80-102) | 92(80-104) | 0.602 | 92(80-106) | 92(80 -105) | 0.898 |
| Mean respiratory rate (/min) | 20(17-24) | 21(18-25) | 0.008 | 21(18-25) | 21(18-25) | 0.727 |
| ARDS severity |  |  | <0.001 |  |  | 0.966 |
| 1 | 187(18.7%) | 44(12.8%) |  | 40(12.7%) | 43(13.7%) |  |
| 2 | 474(47.4%) | 145(42.0%) |  | 137(43.4%) | 141(44.9%) |  |
| 3 | 340(34.0%) | 156 (45.2%) |  | 139(44.0%) | 140(44.6%) |  |
| ICU type |  |  | 0.004 |  |  | 0.148 |
| CCU | 120( 12.0%) | 53( 15.4%) |  | 33(10.4%) | 57(18.2%) |  |
| CSRU | 154( 15.4%) | 26( 7.5%) |  | 40(12.7%) | 26(8.3%) |  |
| MICU | 455 ( 45.5%) | 172( 49.9%) |  | 154(48.7%) | 155(49.4%) |  |
| SICU | 135( 13.5%) | 43( 12.5%) |  | 40(12.7%) | 41(13.1%) |  |
| TSICU | 137 ( 13.7%) | 51( 14.8%) |  | 49(15.5%) | 51(16.2%) |  |
| Admission type |  |  | 0.088 |  |  | 0.370 |
| ELECTIVE | 111 (11.1%) | 24 (7.0%) |  | 32(10.1%) | 24(7.6%) |  |
| EMERGENCY | 851 (85.0%) | 307(89.0%) |  | 267(22.2%) | 286(91.1%) |  |
| URGENT | 39 (3.9%) | 14 (4.0%) |  | 17(5.4%) | 14(4.5%) |  |
| Gender |  |  | 0.825 |  |  | 0.522 |
| Male | 588(58.7%) | 205(59.4%) |  | 197(62.3%) | 194(61.8%) |  |
| Diabetes | 241(24.1%) | 77(22.3%) | 0.528 | 76(24.1%) | 74(23.6%) | 0.718 |
| Hypertension | 311(31.1%) | 94(27.2%) | 0.182 | 92(29.1%) | 85(27.1%) | 0.823 |
| COPD | 158(15.8%) | 43(12.5%) | 0.136 | 44(13.9%) | 42(13.4%) | 0.722 |
| Sepsis | 692(69.1%) | 263(76.2%) | 0.012 | 232(73.4%) | 242(77.1%) | 0.713 |
| CHF | 384 (38.4%) | 126 (36.5%) | 0.544 | 123(38.9%) | 122(38.9%) | 0.741 |
| AFIB | 287(28.7%) | 97(28.1%) | 0.844 | 104(32.9%) | 94(29.9%) | 0.286 |
| Renal | 90(9.0%) | 30(8.7%) | 0.868 | 25(7.9%) | 28(8.9%) | 0.737 |
| Liver | 78(7.8%) | 27(7.8%) | 0.984 | 28(8.9%) | 24(7.6%) | 0.501 |
| CAD | 185(18.5%) | 64(18.5%) | 0.977 | 58(18.4%) | 61(19.4%)） | 0.878 |
| Stroke | 81 (8.1%) | 31(9.0%) | 0.604 | 31(9.8%) | 29(9.2%) | 0.709 |
| Malignancy | 172 (17.2%) | 53(15.4%) | 0.434 | 61(19.3%) | 50(15.9%) | 0.196 |
| Day of ICU admission |  |  | 0.074 |  |  | 0.099 |
| Monday | 166 (16.9%) | 52 (15.0%) |  | 55(17.4%) | 49(15.6%) |  |
| Tuesday | 133(13.2%) | 42(12.7%) |  | 42(13.3%) | 38(12.1%) |  |
| Wednesday | 156 (15.5%) | 61(17.1%) |  | 39(12.3%) | 56(17.8%) |  |
| Thursday | 139 (13.8%) | 37(11.9%) |  | 44(13.9%) | 34(10.8%) |  |
| Friday | 167(16.9%) | 52(15.2%) |  | 53(16.8%) | 50(15.9%) |  |
| Saturday | 123 (11.9%) | 39(12.3%) |  | 46(14.5%) | 38(12.1%) |  |
| Sunday | 117 (11.9%) | 62(15.6%) |  | 37(11.7%) | 59(18.8%) |  |
| Lactic acid |  |  | 0.019 |  |  | 0.931 |
| Missing value | 415(41.5%) | 155(45.0%) |  | 136 (43.0%) | 144(45.6%) |  |
| <4 | 98(9.8%) | 48(13.9%) |  | 43(13.6%) | 44 (14.0%) |  |
| >4 | 488(48.8%) | 142(41.1%) |  | 137(43.4%) | 136(43.3%) |  |
| Vasopressin use (n, %) | 373(37.3%) | 249(72.2%) | <0.001 | 219(69.3%) | 231(73.6%) | 0.134 |

**Abbreviations:**PO_2_=oxygen partial pressure,FiO2=Fraction of inspiration O2, PEEP=positive end expiratory pressure, MAP=Mean arterial pressure, SpO_2_=pulse oxygen saturation, PBW=Parts by Weight, PCO_2=_carbon dioxide partial pressure, SAPS=simplified acute physiology score, SOFA=sequential organ failure assessment score, OASIS=Oxford acute severity of illness score, BUN=blood urea nitrogen, CCU=Coronary Care Unit , CSRU=Cardiovascular Surgery Rehabilitation Unit, MICU=Medical Intensive Care Unit, SICU=Surgical Intensive Care Unit，TSICU=Trauma Surgery Intensive Care Unit, CHF=Congestive heart failure, AFIB=atrial fibrillation, COPD=chronic obstructive pulmonary disease, CAD= coronary artery disease.
